# miR-196a-5p-Rich Extracellular Vesicles from Trophoblasts Induce M1 Polarization of Macrophages in Recurrent Miscarriage

**DOI:** 10.1155/2022/6811632

**Published:** 2022-05-23

**Authors:** Jing Zhang, Yu Tao, Renfei Cai, Yao Wang

**Affiliations:** Department of Assisted Reproduction, The Ninth People's Hospital, Shanghai Jiaotong University School of Medicine, Shanghai, China

## Abstract

**Purpose:**

Numerous studies have described the presence of crosstalk between trophoblasts and macrophages and the critical role it plays in recurrent miscarriage (RM). However, the mechanism of trophoblast-derived extracellular vesicle (EV) miRNAs and their interactions with decidual macrophages in the pathogenesis of RM remains unclear.

**Materials and Methods:**

miRNA-seq was used to identify the differentially expressed miRNAs between RM patients and healthy controls. qPCR and in situ hybridization assays were performed to analyze the expression levels of miR-196a-5p in RM. THP-1 cells were treated with EVs, and qPCR and flow cytometry were performed to explore the polarization of macrophages. To explore the crosstalk between trophoblasts and macrophages, a coculture model and a series of cell function assays were performed.

**Results:**

We first demonstrated that miR-196a-5p expression was higher in the cytotrophoblasts of villous tissues and plasma EVs from RM patients. miR-196a-5p derived from trophoblasts could be transferred into macrophages via EVs to induce M1 polarization via I*κ*B*α*-mediated NF-*κ*B pathway. Moreover, we found that M1 macrophages induced by EV miR-196a-5p derived from trophoblasts conversely regulated the proliferation, migration, and apoptosis of trophoblasts via TNF-*α*.

**Conclusions:**

This study indicated that trophoblast-derived EV miR-196a-5p was positively associated with RM and functioned by regulating the crosstalk between trophoblasts and macrophages. These findings may attribute to identify a novel biomarker specific for RM.

## 1. Introduction

Recurrent miscarriage (RM) is defined as two or more consecutive pregnancy loss before 20 weeks of gestation and occurs in approximately 2-5% of childbearing couples [1, 2]. Numerous studies have indicated that trophoblasts play a critical role in embryo implantation and the formation of a healthy maternal-fetal interface [3]. Factors that impair trophoblast function and disrupt the balance in the maternal-fetal interface may cause a series of pregnancy complications, including preeclampsia, fetal intrauterine growth restriction, and RM [4]. Trophoblast function is regulated by key genes that mediate the crosstalk between trophoblasts and the placental microenvironment, although the mechanism is complex and incompletely understood.

It has been reported that extracellular vesicles (EVs) have two primary subtypes: endosome-origin small EVs and plasma membrane-derived ectosomes, with a diameter ranging from 40-160 nm and 50-1000 nm, respectively [5, 6]. EV contains a variety of bioactive substances, such as miRNAs, lncRNAs, and proteins, which mediate the interactions between cells [5, 6]. EV-derived miRNAs can be transported into nearby or distant cells, and they subsequently modulate the recipient cells. miRNAs are 17-24 nt length small noncoding RNAs and regulate the mRNA expression of target genes by binding to their 3′-untranslated regions (3′-UTRs) [7]. An increasing number of studies have revealed that EVs play critical roles in regulating the embryo implantation and immune balance at the maternal-fetal microenvironment [8, 9]. For example, decidual macrophage-derived EV miR-153-3p from unexplained RM patients suppressed the proliferative and migration abilities of trophoblast cells via regulation of the IDO/STAT3 pathway [10]. It was reported that trophoblast-derived EVs could recruit and induce monocytes to secrete cytokines, such as G-CSF, IL-6, and TNF-*α*, which is important for embryo implantation, stromal remodeling, and angiogenesis [11]. However, the communication mechanisms between trophoblasts and macrophages mediated by EVs require further study.

Macrophages account for around 20-30% of decidual immune cells and are crucial in the modulation of the decidual immune microenvironment [12]. Macrophages are divided into subtypes: the proinflammatory M1 subtype with and the anti-inflammatory M2 subtype. Numerous studies have indicated that the proportion of M1/M2 macrophages in the decidual tissue of patients with unexplained RM was significantly increased compared with women who had healthy pregnancies in first trimester [13, 14]. It has been reported that M1 macrophages can affect the invasion and migration of trophoblasts, and this may result in pregnancy failure [15, 16]. Although the crosstalk between the maternal-fetal microenvironment and macrophages has been extensively researched, the underlying mechanisms of decidual macrophage M1 polarization have not been elucidated [12].

In this study, we discovered that miR-196a-5p may be associated with RM using miRNA-seq analysis. We further found that miR-196a-5p could be transferred into macrophages via EVs to induce M1 polarization via activation of the I*κ*B*α*-mediated NF-*κ*B pathway. Functionally, we found M1 macrophages induced by trophoblasts released EVs rich in miR-196a-5p, and this conversely regulated trophoblast function. Together, these results confirmed the existence of a mean of communication between trophoblasts and M1 macrophages and identified a potential mechanism by which this communication occurred. This study may assist in identifying a novel biomarker specific for RM.

## 2. Methods

### 2.1. Patient Samples

A total of 60 cases of villous tissues and plasma samples were obtained from 30 RM patients (age 24–35, mean age 30.75 ± 3.14 years) and 30 healthy pregnant women (age 24–35, mean age 29.25 ± 2.86 years) who had been treated at the Ninth People's Hospital Affiliated to Shanghai Jiaotong University School of Medicine during January to December 2020. The exclusion criteria for recruitment were as follows: (1) parents or an abortus with an abnormal karyotype, mothers with (2) an abnormal uterine anatomy, (3) abnormal immune function, (4) endocrine or metabolic disorders, (5) infectious diseases, or (6) cases with other identified causes of miscarriage. The healthy control samples (HC) were collected from women with a previous normal pregnancy and no history of miscarriage, preterm labor, or preeclampsia. These individuals terminated the unwanted pregnancies by artificial abortion. All the samples were collected at 6-12 weeks of gestation. The present study was approved by the Medical Ethics Committee of the Ninth People's Hospital Affiliated to Shanghai Jiaotong University School of Medicine.

### 2.2. RNA Extraction and Reverse Transcription-Quantitative PCR

Total RNA extraction was performed using TRIzol® reagent (Thermo Fisher Scientific, Inc.). Reverse transcription and quantitative PCR was conducted using a PrimeScript RT reagent kit (Takara Bio, Inc.) and SYBR Premix Ex Taq (Takara Bio, Inc.), respectively. Relative mRNA expression was calculated according to the 2^–*ΔΔ*Cq^ method. U6 or GAPDH was used as loading controls. The sequences of the primers used in this study are described in Supplementary Table S1.

### 2.3. Western Blotting

Samples were prepared using radioimmunoprecipitation assay buffer and then centrifuged at 14,000 × g for 10 min at 4°C. 10% SDS-PAGE was used to separate. The protein lysates (10 *μ*g) according to the molecular weight. After separation, proteins were transferred to polyvinylidene fluoride (PVDF) membranes (Millipore Sigma). Subsequently, PVDF membranes were incubated with primary antibodies (Supplementary Table S2) at 4°C overnight. Then, secondary antibodies were used to treat with the membrane at room temperature for 1 h. The signals were detected with an enhanced chemiluminescence reagent (Shanghai Yeasen Biotechnology Co., Ltd.) according to the manufacturer's instructions.

### 2.4. In Situ Hybridization

The expression feature of miR-196a-5p in 60 cases of human villous tissues was examined using in situ hybridization as described previously [17]. Positive staining was detected using a diaminobenzidine substrate kit (Changsha AxyBio Biotech, Co., Ltd.).

### 2.5. Cell Culture

The HTR-8/SVneo cells and the human monocytic THP-1 cells were obtained from The Cell Bank of Type Culture Collection of the Chinese Academy of Sciences. Cells were cultured in 10% FBS RPMI 1640 medium and incubated in a humidified incubator with 5% CO_2_ at 37°C. To induce differentiation into macrophages, THP-1 cells (1 × 10^6^) were treated with phorbol 12-myristate 13-acetate (PMA; Abcam) at the concentration of 100 ng/ml for 48 h.

HTR-8 cells and THP-1 cells (EV-pretreated) were cocultured using Transwell inserts (pores 0.4 *μ*m; Millipore Sigma) in a 12-well plate for 48 h. Cell functions were analyzed using CCK-8, Transwell, and flow cytometry assays.

### 2.6. Culture Medium (CM) Preparation

Approximately 2 × 10^6^ HTR-8 cells were incubated in 10 cm dishes for 24 h. Then, the CM was replaced with serum-free medium, and cells were further incubated for 48 h. CM was collected and spun down at 3,000 × g for 10 min and stored at 4°C. To ensure depletion of EVs from the CM, the CM was centrifuged successively at 300 × g for 20 min, 2,000 × g for 20 min, and 12,000 × g for 70 min at 4°C.

### 2.7. EV Isolation and Transmission Electron Microscopy

For EV isolation, CM was filtered by a PVDF filter (0.22 *μ*m, Millipore Sigma). Differential centrifugation was used to isolate EVs as previously described [18]. The size and concentration of the EVs were detected with a NanoSight NS300 instrument and analyzed using the nanoparticle tracking analysis (NTA) 3.0 software (Malvern Instruments Ltd., UK). ExoQuick Plasma prep and Exosome precipitation kit (SBI, USA) was used to obtain the plasma EVs according to the instructions.

For transmission electron microscopy detection, EVs were dropped onto the copper grid. EVs were incubated with 2% phosphotungstic acid for 2 min to negatively staining and then air-dried for 15 min. A transmission electron microscope (FEI Tecnai G2 Spirit; Thermo Fisher Scientific, Inc.) running at 80 kV was used to observe the EVs.

### 2.8. Fluorescently Labeled EV Transfer Assay

The EVs isolated from HTR-8 cells were marked with the lipophilic dye, DiO (10 *μ*M, Thermo Fisher Scientific, Inc.), for 20 min at 37°C. DiO-labeled EVs were added into the culture medium of macrophages at the concentration of 25 *μ*g/ml for 24 h. After staining of the cytoskeleton with FITC Phalloidin (Shanghai Yeasen Biotechnology Co., Ltd.), the internalization of EVs was assessed using a confocal microscope.

### 2.9. Plasmid Construction and Cell Transfection

miR-196a-5p mimics, inhibitors, and negative controls were purchased from Guangzhou RiboBio Co., Ltd. si-hnRNPA1, the negative control siRNA, I*κ*B*α* overexpression plasmid, and a control vector were supplied by Shanghai GenePharma Co., Ltd. Transfection of plasmids was performed using Lipofectamine® 3000 reagent (Invitrogen; Thermo Fisher Scientific, Inc.). Transfection of siRNAs or miRNA mimics or inhibitors was performed using Lipofectamine® RNAiMAX (Invitrogen; Thermo Fisher Scientific, Inc.) at a final concentration of 100 nM. The primers used for plasmid construction and the sequences of the siRNAs and miRNA mimics used in this study are stated in Supplementary Table S3.

### 2.10. Luciferase Reporter Assay

Luciferase reporter plasmids containing the 3′-UTR I*κ*B*α*, with either wild-type or a mutated miR-196a-5p binding site, were generated by Shanghai GeneChem Co., Ltd. Luciferase reporter plasmids (200 ng/well), miR-196a-5p mimics or miR-196a-5p inhibitors (200 ng/well), and Renilla luciferase vector (10 ng) were cotransfected into 293T cells using Lipofectamine® 3000. After transfection for 48 h, a Dual-Luciferase Reporter assay kit was used to detect the luciferase and *Renilla* activity based on the manufacturer's instructions (Promega Corporation).

### 2.11. Biotin-Labeled RNA Pull down

The nuclear and cytoplasmic lysates were extracted using a NE-PERTM nuclear and cytoplasmic extraction kit (Thermo Fisher Scientific, Inc.). After modified with biotin, a total of 100 pmol wild-type or mutated miR-196a oligonucleotides were added to the lysates for 12 h at 4°C. Then, agarose beads (Invitrogen; Thermo Fisher Scientific, Inc.) were added into the lysates. After incubation at 4°C for 4 h, the proteins were extracted and analyzed by western blotting. Poly (G) (5′-GGGGGGGGGGGGGGGGGGGGG-3′) was labeled with biotin and used as a negative control.

### 2.12. RNA-Binding Protein Immunoprecipitation (RIP) Assay

RIP assays were conducted with a Magna RIP™ kit (Millipore Sigma) according to the manufacturer's instructions. Briefly, formaldehyde cross-linked cells (1 × 10^7^) were lysed in lysis buffer. After centrifugation at 14,000 × g for 15 min, 3 *μ*g anti-hnRNPA1 antibody (Cell Signaling Technology, Inc.) or IgG (Cell Signaling Technology, Inc.) was added the lysate (1 mg), respectively. The solutions were stored at 4°C with end-over-end rotation overnight. mirVana™ PARIS™ kit (Ambion; Thermo Fisher Scientific, Inc.) was applied to isolate miRNAs. After reverse transcription, quantitative PCR was performed to measure miR-196a-5p expression.

### 2.13. Immunoprecipitation of miRNA Targets

miR-196a-5p or miR-NC (containing biotin) was transfected into PMA-pretreated THP-1 cells (5 × 10^6^) for 48 h. Then, the cells were lysed. Streptavidin-Dyna beads (50 *μ*l/sample; Invitrogen; Thermo Fisher Scientific, Inc.) were added into the samples. The solutions were rotated overnight at 4°C, and the beads were collected. mirVana™ PARIS™ kit was used to obtain RNA from the beads. Then, samples were reverse transcribed and assessed using quantitative PCR to measure RNA expression levels.

### 2.14. Cell Proliferation Assay

HTR-8 cells (2 × 10^3^ cells per well) were seeded in 96-well plates. CCK-8 solution (10 *μ*l per well; Shanghai Yeasen Biotechnology Co., Ltd.) was added to cells after 0, 24, 48, or 72 h and incubated for a further 2 h. A microplate reader was used to detect the optical density value at 450 nm.

### 2.15. Transwell Assay

Transwell assays were conducted using a Transwell chamber (pores 0.8 *μ*m, Merck Millipore, USA) in a 24-well plate. The top chambers were plated with HTR-8 cells (1 × 10^5^ cells), and to the bottom chambers, CM supplemented with 20% FBS was added. After culturing for 24 h, cells were fixed with paraformaldehyde (4%) and stained with crystal violet.

### 2.16. Flow Cytometry Analysis

For identification of macrophages, CD86-positive cells were detected through flow cytometry. Macrophages were harvested and resuspended in flow cytometry buffer (1x PBS buffer containing 1% BSA) and stained with an anti-CD86 antibody for 30 min at room temperature. Cells were washed with the washing buffer and resuspended in flow cytometry buffer and then analyzed by flow cytometer (BD Biosciences, USA). The FlowJo 7.6.1 software was applied to analyze the data.

Apoptosis assay was performed with an Annexin V-FITC/PI Apoptosis kit (Beyotime Institute of Biotechnology) based on the instructions. Flow cytometry was used to analyze the FITC and PI positive rate.

### 2.17. ELISA

To measure the protein levels of iNOS, IL-1*β*, and TNF-*α* in the CM of macrophages, ELISA was performed using Human iNOS, IL-1*β*, and TNF-*α* ELISA kits (Abcam), based on the manufacturer's instructions. The absorbance was detected at 490 nm using an ELISA plate reader within 30 min of stopping the reactions.

### 2.18. Statistical Analysis

All statistical analysis in this study was performed using statistical software SPSS 21.0 (IBM Corp.). The differences between two groups or multiple groups were analyzed with an unpaired Student's *t*-test or a one-way ANOVA, respectively. All data were obtained from three independent experiments and are presented as the mean ± standard deviation. *p* < 0.05 was considered to indicate a statistically significant difference.

### 2.19. Role of the Funding Source

The study was supported by Seed Founding of Shanghai Ninth People's Hospital, Shanghai Jiaotong University School of Medicine, Shanghai Sailing Program, and the National Nature Science Foundation of China. The funders did not participate in study design, data collection, data analyses, interpretation, or writing.

## 3. Results

### 3.1. miR-196a-5p Is Upregulated in the Cytotrophoblasts (CTBs) of RM Villous Tissues

**Figure 1:**
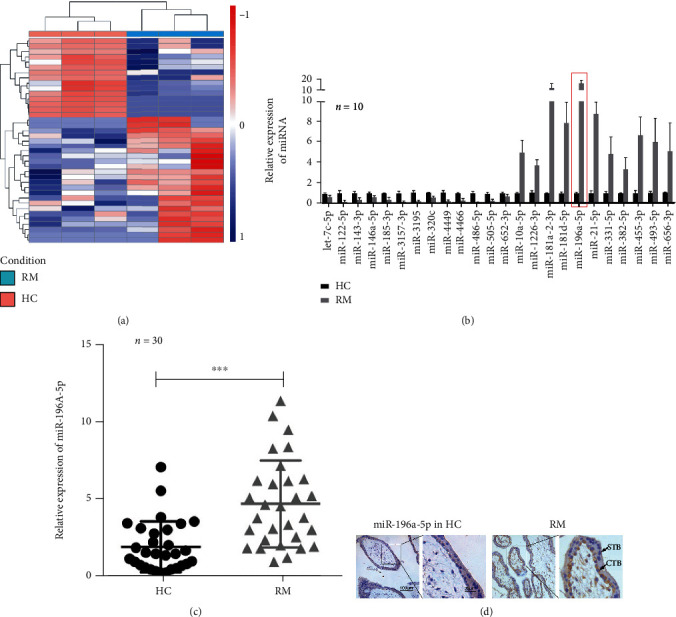
miR-196a-5p expression is upregulated in CTBs of RM villous tissues. (a) Heatmap showing differentially expressed miRNAs between the villous tissues of three RM and three HC. (b) qPCR comparing the expression levels of 24 differentially expressed miRNAs between villous tissues of 10 RM and 10 HC cases. (c) qPCR analysis of the miR-196a-5p expression levels in the villous tissues of 30 RM and 30 HC cases. (d) Representative images of in situ hybridization staining of miR-196a-5p in RM and HC samples. CTBs and syncytiotrophoblasts are indicated by arrows (^∗∗∗^*p* < 0.001). RM: recurrent miscarriage; CTB: cytotrophoblast; HC: healthy control.

To explore the underlying pathogenesis of RM, we performed miRNA-seq on the villous tissues from three RM patients and three HC individuals. A total of 24 differentially expressed miRNAs (consisting of 11 upregulated and 13 downregulated miRNAs) were identified (Figure 1(a) and Figure S1). We further analyzed the expression levels of these 24 miRNAs amongst the tissues from 10 RM patients and 10 HC individuals using qPCR. We found that miR-196a-5p was the top most upregulated miRNA (Figure 1(b)). Subsequently, qPCR was performed to compare miR-196a-5p expression between 30 cases of RM and HC villous tissues and found that the expression level of miR-196a-5p was obviously higher in the RM group than the HC group (Figure 1(c)). To further study the expression features of miR-196a-5p, we performed in situ hybridization assay on 30 samples of RM and HC villous tissues (Figure 1(d)). We found that miR-196a-5p was primarily expressed in the cytoplasm of CTBs. These data revealed that miR-196a-5p was upregulated in the CTBs of RM tissues and may be associated with the pathogenesis of RM.

### 3.2. miR-196a-5p Is Encapsulated within Trophoblast-Derived EVs

**Figure 2:**
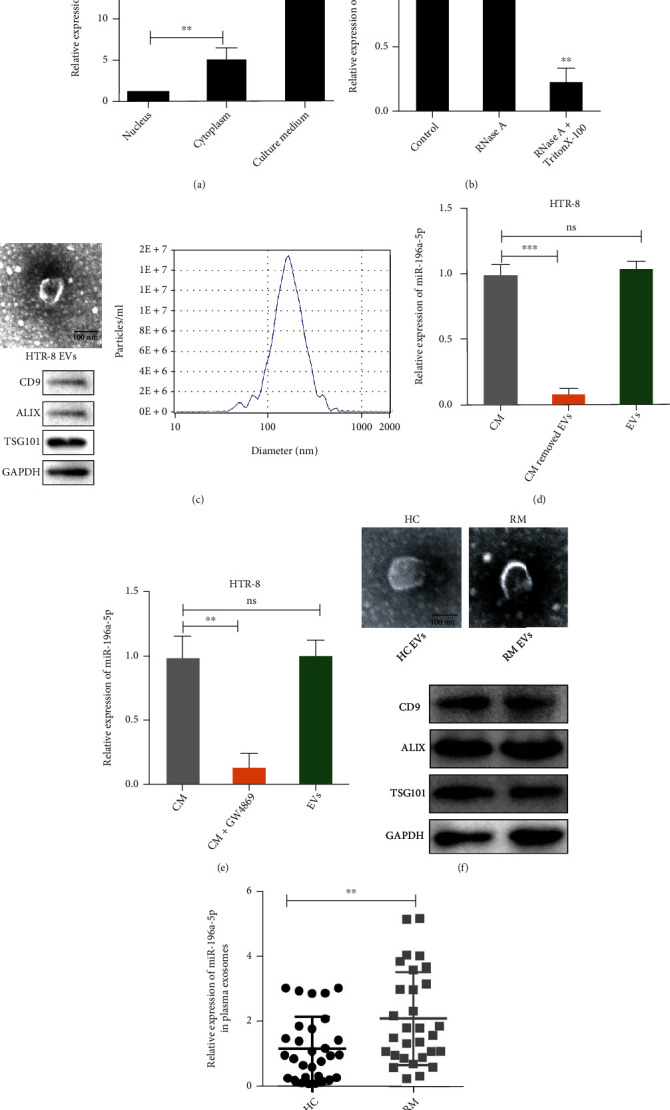
miR-196a is highly expressed in trophoblast-derived EVs and plasma EVs of RM patients. (a) qPCR analysis of the expression levels of miR-196a-5p in the nucleus, cytoplasm, and CM of the HTR-8 cell lines. (b) qPCR analysis of the expression levels of miR-196a-5p in the CM of HTR-8 cells treated with RNase-A (2 mg/ml) alone or in combination with 0.1% Triton X-100. (c) Identification of the EVs derived from HTR-8 cells using transmission electron microscopy (top left), western blotting (bottom left), and NTA (right). (d) qPCR analysis of miR-196a-5p expression levels in the CM, CM with EVs removed by ultracentrifugation, and EVs of HTR-8 cells. (e) qPCR analysis of miR-196a-5p expression in the CM of HTR-8 cells treated with or without GW4869, an inhibitor of EV secretion, and in the EVs derived from the treated and untreated HTR-8 cells. (f) Identification of EVs isolated from the plasma of HC and RM patients through transmission electron microscopy (top) and western blotting (bottom). (g) qPCR analysis comparing the expression levels of miR-196a-5p in the plasma EVs of 30 RM patients and 30 HC individuals (^∗^*p* < 0.05; ^∗∗^*p* < 0.01; ^∗∗∗^*p* < 0.001; ns: not significant). CM: culture medium; NTA: nanoparticle tracking analysis.

To further understand the role of miR-196a-5p in the pathogenesis of RM, we analyzed the expression of miR-196a-5p in the nucleus, cytoplasm, and the CM in HTR-8 human trophoblastic cells. The results demonstrated that miR-196a-5p exhibited the highest expression levels in the cytoplasm and the lowest levels in the nucleus (Figure 2(a)). In addition, miR-196a-5p expression in the CM could not be degraded with RNase-A but could be degraded with RNase-A plus TritonX-100, suggesting that extracellular miR-196a-5p may be packaged into a membranous structure (Figure 2(b)). Recently, it has been reported that EVs participate in intercellular communication in the maternal-fetal interface [7]. Therefore, we speculate miR-196a-5p may be encapsulated in EVs when released from trophoblasts. The EVs from HTR-8 cells were obtained by differential centrifugation and then confirmed with electron microscopy, NTA, and western blotting analyses of EV markers (Figure 2(c)). To further identify whether miR-196a-5p derived from EVs, we removed EVs by ultracentrifugation or treatment with EV secretion inhibitor GW4869 and analyzed miR-196a-5p expression in the CM, CM without EVs, and in the EVs themselves. The results demonstrated that the expression levels of miR-196a-5p in CM were increased when compared with that in the CM depleted of EVs by ultracentrifugation and treatment with GW4869 but did not demonstrate a significant difference when compared with the expression in EVs (Figures 2(d) and 2(e)). In addition, we isolated EVs from the plasma of HC and RM patients (Figure 2(f)). We also found the miR-196a-5p expression in plasma EVs of RM patients was higher than that of HC individuals (Figure 2(g)). These results suggested that miR-196a-5p was primarily packaged within trophoblast-derived EVs.

### 3.3. Trophoblast-Derived EV miR-196a-5p Promotes M1 Polarization of Macrophages

**Figure 3:**
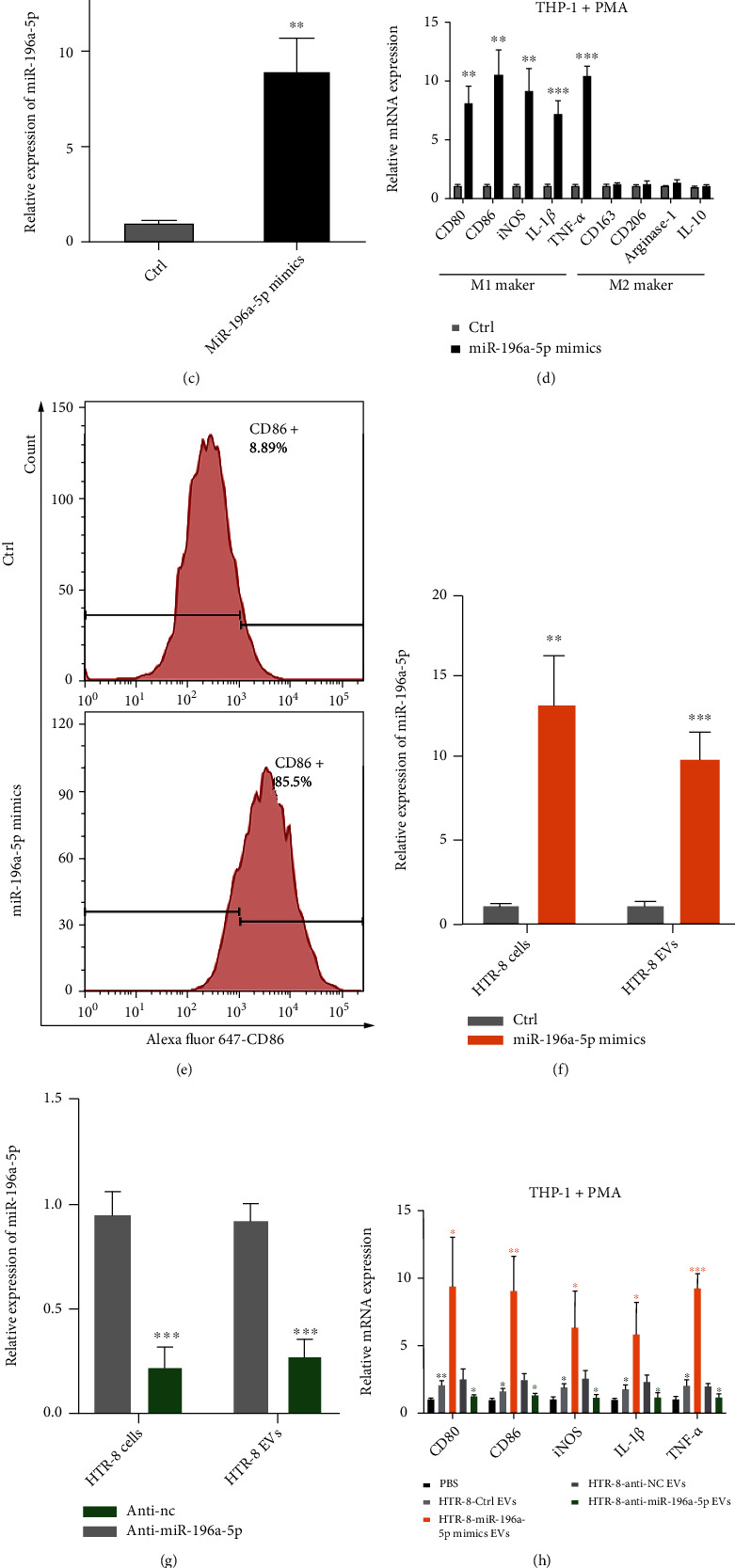
Trophoblast-derived EV miR-196a-5p promotes M1 polarization of macrophages. (a) Morphology of THP-1 cells treated with PMA for 24 h (left) and qPCR analysis of the expression levels of the macrophage marker, CD68 (right). (b) Immunofluorescence images demonstrating the internalization of DiO-labeled HTR-8-derived EVs (green) by PMA-treated THP-1 cells. (c, d) PMA-pretreated THP-1 cells were transfected with miR-196a-5p mimics, and (c) the expression of miR-196a-5p and (d) the expression of typical markers of M1 and M2 macrophages were determined using qPCR. (e) CD86-positive cell count (M1 marker) in the PMA-pretreated THP-1 cells after transfection with miR-196a-5p mimics was detected using flow cytometry. (f, g) HTR-8 cells were transfected with miR-196a-5p mimics, anti-miR-196a-5p, or their respective controls, and the expression levels of miR-196a-5p in HTR-8 cells and HTR-8 derived-EVs were measured using qPCR. PMA-pretreated THP-1 cells were treated with EVs derived from the miR-196a-5p overexpressing or knockdown HTR-8 cells, as well as from their respective controls. (h) qPCR analysis of the expression levels of M1 markers (black^∗^: HTR-8-Ctrl EVs vs. PBS; yellow: HTR-8-miR-196a-5p mimics EVs vs. HTR-8-Ctrl EVs; green^∗^: HTR-8-anti-miR-196a-5p EVs vs. HTR-8-anti-NC EVs). (i) CD86-positive cell count was determined using flow cytometry (^∗^*p* < 0.05; ^∗∗^*p* < 0.01; ^∗∗∗^*p* < 0.001). EV: extracellular vesicle; PMA: phorbol 12-myristate 13-acetate.

Macrophages are one of the most important types of immune cells at the maternal-fetal interface [19]. An increasing number of studies have shown that EV miRNAs can induce polarization of macrophages in the tumor microenvironment [20]. To explore whether trophoblast-derived EVs could regulate the polarization of macrophages, THP-1 cells were induced as M0 macrophages using PMA and incubated with EVs derived from HTR-8 cell (DiO labeled). qPCR verified that the expression of M0 marker CD68 in THP-1 cells was upregulated after treatment with PMA (Figure 3(a)). The confocal analysis showed internalization of DiO-labeled HTR-8 derived EVs in PMA-pretreated THP-1 cells. To understand the role of miR-196a-5p on polarization of macrophages, PMA-pretreated THP-1 cells were transfected with miR-196a-5p mimics (Figure 3(c)). The typical markers of macrophages were measured using qPCR, and the results showed that markers of M1 macrophages (CD80, CD86, IL-1*β*, and TNF-*α*), not M2 macrophages, were significantly upregulated in PMA-pretreated THP-1 cells after transfection with miR-196a-5p mimics (Figure 3(d)). Additionally, the number of CD86-positive cells was increased in PMA-pretreated THP-1 cells after overexpression of miR-196a-5p (Figure 3(e)). We further constructed miR-196a-5p overexpressing or knockdown HTR-8 cells. EVs were isolated from these cells and incubated with PMA-pretreated THP-1 cells. qPCR analysis showed that the expression of miR-196a-5p was, respectively, increased or decreased in EVs derived from miR-196a-5p-overexpressing HTR-8 cells or miR-196a-5p knockdown HTR-8 cells compared with their respective controls (Figures 3(f) and 3(g)). Subsequently, PMA-pretreated THP-1 cells were incubated with PBS or EVs derived from miR-196a-5p-overexpressing HTR-8 cells, miR-196a-5p knockdown HTR-8 cells, or control cells. We found that the expression of M1 markers in PMA-pretreated THP-1 cells was increased after incubating with HTR-8 control cell-derived EVs (Figures 3(h) and 3(i)). Additionally, incubation with EVs derived from miR-196a-5p-overexpressing HTR-8 cells or miR-196a-5p knockdown HTR-8 cells could promote or inhibit expression of M1 markers, respectively (Figures 3(h) and 3(i)). Together, these results indicated that trophoblast-derived EV miR-196a-5p could induce M1 polarization of macrophages.

### 3.4. hnRNPA1 Mediates miR-196a-5p Packaging into Trophoblast-Derived EVs

**Figure 4:**
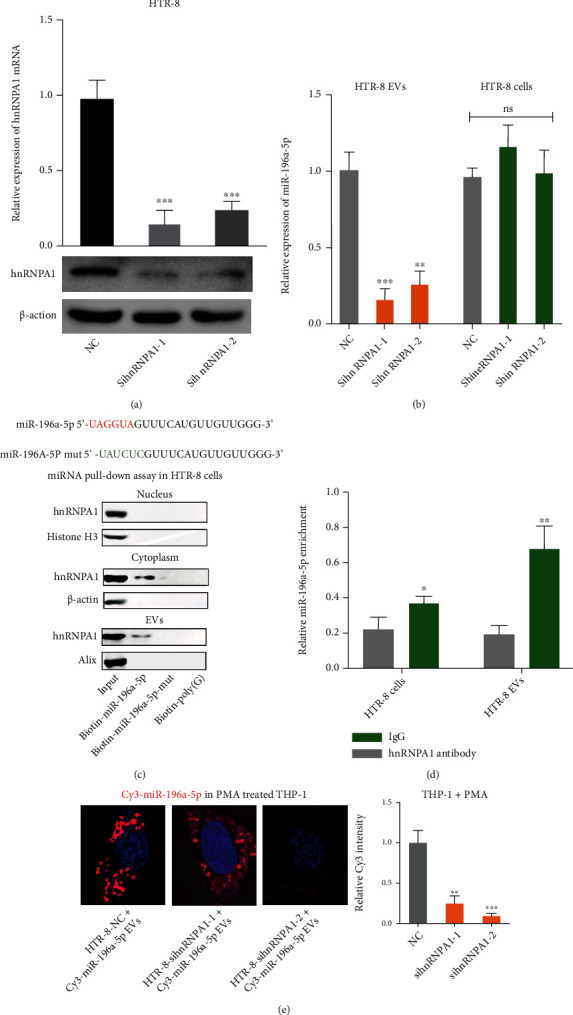
hnRNPA1 regulates miR-196a-5p packaging into EVs from trophoblasts. (a) The mRNA and protein expression levels of hnRNPA1 were evaluated by qPCR and western blotting in HTR-8 cells transfected with si-hnRNPA1 as well as the control siRNA. (b) qPCR analysis of miR-196a-5p expression levels in HTR-8 cell-derived EVs and the HTR-8 cells after transfection with si-hnRNPA1. (c) miRNA pull-down assays were performed using the nuclear, cytoplasmic, or EV lysates of HTR-8 cells. The expression of hnRNPA1 pulled by biotin-labeled miR-196a or mutant miR-196a was analyzed using western blotting. (d) RIP assays with hnRNPA1 antibody or IgG were performed using the lysates of HTR-8 cells or the EVs. (e) Cy3-miR-196a-5p and si-hnRNPA1 were cotransfected into HTR-8 cells for 48 h. Then, EVs were isolated and added to the PMA-pretreated THP-1 cells. Fluorescence microscopy was used to evaluate the red fluorescent signals in THP-1 cells (^∗^*p* < 0.05; ^∗∗^*p* < 0.01; ^∗∗∗^*p* < 0.001; ns: not significant). EV: extracellular vesicle; RIP: RNA immunoprecipitation.

It has been reported that the packaging of RNA into EVs requires an RNA-binding protein for transport [21]. The hnRNPA1 protein can mediate miR-196a packaging into cancer-associated fibroblast-derived EVs [22]. We predicted the potential RNA-binding proteins associated with miR-196a-5p using the RNA-binding protein database specificities (RBPDB, http://rbpdb.ccbr.utoronto.ca/) (Figure S2(a)). We found that hnRNPA1, ZRANB2, and ELAVL1 were the three most correlated. To explore whether hnRNPA1, ZRANB2, or ELAVL1 participated in the transfer of trophoblast-derived EV miR-196a-5p to macrophages, HTR-8 cells were transfected with hnRNPA1-, ZRANB2-, or ELAVL1-specific siRNAs (Figure 4(a); Figure S2(b)-(c)). The miR-196a-5p expression levels of HTR-8 derived EVs and HTR-8 cells were evaluated. We found that knockdown of hnRNPA1 could suppress the expression of miR-196a-5p in EVs but did not affect its expression in HTR-8 cells; knockdown of ZRANB2 or ELAVL1 could not change miR-196a-5p expression both in HTR-8 derived EVs and HTR-8 cells (Figure 4(b); Figure S2(d)-(e)). Besides, hnRNPA1 could specifically bind to the UAGGUA sequence of miR-196a-5p according to the RBPDB (Figure S2). We constructed biotin-labeled miR-196a-5p with wild-type or mutated hnRNPA1 binding sites. The RNA pull-down assays were performed, and we observed the interaction between hnRNPA1 and miR-196a-5p in the cytoplasm and EVs, but not in the nucleus; hnRNPA1 could not bind to the mutant miR-196a-5p (Figure 4(c)). Additionally, RIP assays in the cell and EV lysates of trophoblasts demonstrated the enrichment of miR-196a-5p was increased in the hnRNPA1 antibody group compared with the IgG group (Figure 4(d)). To further verify the transfer mechanism of miR-196a-5p, we established a coculture model with PMA-pretreated THP-1 cells and HTR-8 cells that had been cotransfected with Cy3-labeled miR-196a-5p and hnRNPA1 siRNA. The confocal analysis showed that knockdown of hnRNPA1 could reduce the transfer of miR-196a-5p from trophoblasts to PMA-pretreated THP-1 cells via EVs (Figure 4(e)). In addition, the correlation analysis showed that the expression of hnRNPA1 in 30 cases RM villous tissues was positively associated with the expression of miR-196a-5p (Figure S4). Taken together, the above results showed that hnRNPA1 mediates the transfer of EV miR-196a-5p to macrophages.

### 3.5. Trophoblast-Derived EV miR-196a-5p Promotes M1 Polarization of Macrophages via the I*κ*B*α*-Mediated NF-*κ*B Pathway

**Figure 5:**
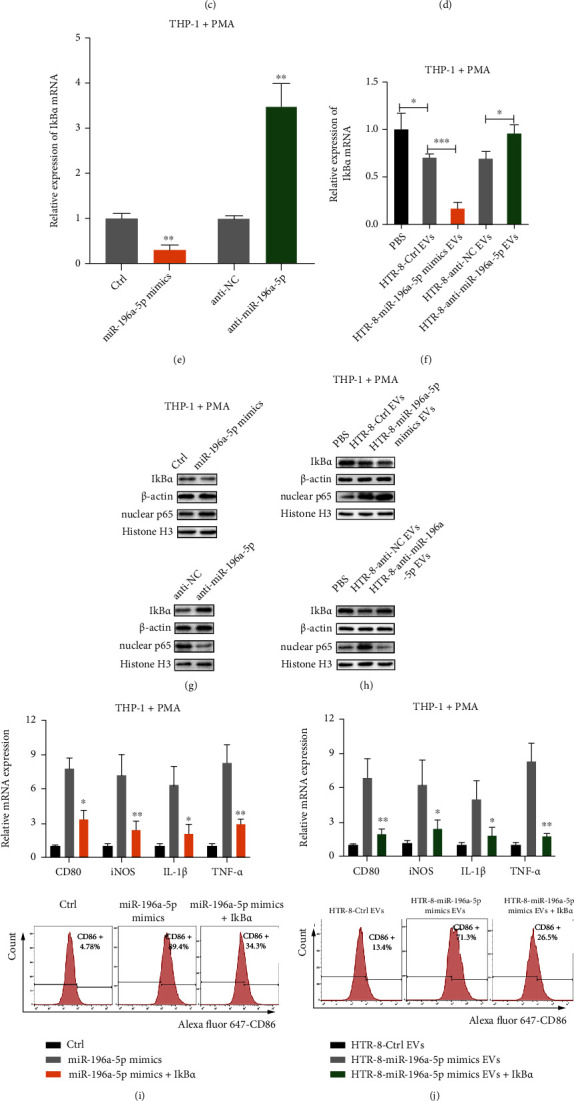
Trophoblast-derived EV miR-196a-5p promotes M1 polarization of macrophages via an I*κ*B*α*-mediated NF-*κ*B pathway. (a) Predicted miR-196a-5p binding sequence on the 3′-UTR of I*κ*B*α*. (b) The effects of overexpression or knockdown of miR-196a-5p on the luciferase activity of I*κ*B*α* reporter gene in 293T cells. (c) Luciferase activity of I*κ*B*α* reporter in 293T cells treated with EVs (25 *μ*g/ml) derived from miR-196a-5p overexpressing or knockdown HTR-8 cells. (d) PMA-pretreated THP-1 cells were transfected with biotin-labeled miR-NC or miR-196a-5p for 48 h. Interaction of I*κ*B*α* transcripts with miR-196a-5p was analyzed using a pull-down assay. (e) qPCR analysis of I*κ*B*α* expression levels in the PMA-pretreated THP-1 cells after transfecting with miR-196a-5p mimics or anti-miR-196a-5p. (f) The changes in I*κ*B*α* mRNA expression in PMA-pretreated THP-1 cells treated with EVs (25 *μ*g/ml) derived from miR-196a-5p overexpressing or knockdown HTR-8 cells or their respective controls. (g) Western blotting analysis of the expression levels of I*κ*B*α* and nuclear p65 in PMA-pretreated THP-1 cells after transfection with miR-196a-5p mimics or anti-miR-196a-5p. (h) Western blot analysis of the expression levels of I*κ*B*α* and nuclear p65 in PMA-pretreated THP-1 cells treated with EVs (25 *μ*g/ml) derived from miR-196a-5p overexpressing or knockdown HTR-8 cells. (i–k) PMA-treated THP-1 cells were treated with miR-196a-5p mimics or EVs derived from miR-196a-5p-overexpressing HTR-8 cells and further transfected with an I*κ*B*α* overexpression plasmid. (i, j) The expression levels of typical M1 markers were measured using qPCR and flow cytometry. (k) The protein expression levels of I*κ*B*α* and nuclear p65 were measured using western blotting. (l) PMA-treated THP-1 cells were treated with EVs derived from miR-196a-5p-overexpressing HTR-8 cells and further treated with BAY11-7085, an inhibitor of the NF-*κ*B pathway. The expression of typical M1 markers was measured using qPCR and flow cytometry (^∗^*p* < 0.05; ^∗∗^*p* < 0.01; ^∗∗∗^*p* < 0.001; ns: not significant). EV: extracellular vesicle; PMA: phorbol 12-myristate 13-acetate.

As reported previously, the NF-*κ*B pathway is involved in the polarization of macrophages [23, 24]. Interestingly, we analyzed the http://microrna.org database and found that I*κ*B*α* may be a downstream target of miR-196a-5p (Figure 5(a)). To verify whether miR-196a-5p regulates the NF-*κ*B pathway via targeting I*κ*B*α* expression, a luciferase reporter construct of the I*κ*B*α* 3′-UTR, containing a wild type or mutated miR-196a-5p binding site was constructed and cotransfected into 293T cells with miR-196a-5p mimics or anti-miR-196a-5p. The luciferase activity was decreased or increased after cotransfection of 293T cells with miR-196a-5p mimics or anti-miR-196a-5p with the wild-type I*κ*B*α* 3′-UTR construct, respectively, but not the mutant construct (Figure 5(b)). In addition, HTR-8 derived EVs suppressed the luciferase activity of I*κ*B*α* 3′-UTR in 293T cells; the luciferase activity was decreased and increased when 293T cells were treated with EVs derived from miR-196a-5p overexpressing and knockdown HTR-8 cells, respectively, when compared with their respective paired controls (Figure 5(c)). To study the association between miR-196a-5p with I*κ*B*α*, a biotin-labeled miRNA pull-down assay was performed [25]. Interestingly, the enrichment of I*κ*B*α* mRNA was increased in PMA-pretreated THP-1 cells transfected with biotin-labeled miR-196a-5p (Figure 5(d)). Additionally, qPCR assays showed that overexpression or knockdown of miR-196a-5p could downregulate or upregulate I*κ*B*α* mRNA expression in PMA-pretreated THP-1 cells, respectively (Figure S3, Figure 5(e)). I*κ*B*α* mRNA expression in PMA-pretreated THP-1 cells was decreased after incubation with the EVs derived from HTR-8 control cells (Figure 5(f)). Moreover, incubation with EVs derived from miR-196a-5p-overexpressing HTR-8 cells or miR-196a-5p knockdown HTR-8 cells could inhibit or promote I*κ*B*α* mRNA expression, respectively (Figure 5(f)). Consistently, the protein levels of I*κ*B*α* and nuclear p65 were also regulated by miR-196a-5p and EV miR-196a-5p (Figures 5(g) and 5(h)). These results indicated that EV miR-196a-5p could downregulate I*κ*B*α* expression by targeting its 3′-UTR and activating the NF-*κ*B pathway in PMA-treated THP-1 cells.

To study the combined effect of miR-196a-5p and I*κ*B*α* on the polarization of macrophages, PMA-treated THP-1 cells were treated with miR-196a-5p mimics or EVs derived from miR-196a-5p-overexpressing HTR-8 cells and further transfected with the I*κ*B*α* overexpression plasmid. The results showed that the upregulation of M1 markers induced by miR-196a-5p mimics or EV miR-196a-5p both could be attenuated by overexpression of I*κ*B*α* (Figures 5(i) and 5(j)). I*κ*B*α* overexpression also suppressed the activation of the NF-*κ*B pathway caused by miR-196a-5p mimics or EV miR-196a-5p (Figure 5(k)). Notably, BAY11-7085 (an inhibitor of NF-*κ*B pathway) partially blocked M1 polarization induced by EV miR-196a-5p derived from HTR-8 cells. Thus, we confirmed that trophoblast-derived EV miR-196a-5p promotes M1 polarization of macrophages via downregulation of I*κ*B*α* and activation of the NF-*κ*B pathway.

### 3.6. Trophoblast-Derived EV miR-196a-5p Induces M1 Polarization of Macrophages to Suppress Proliferation and Invasion and Promote Apoptosis of HTR-8 Cells in Return

**Figure 6:**
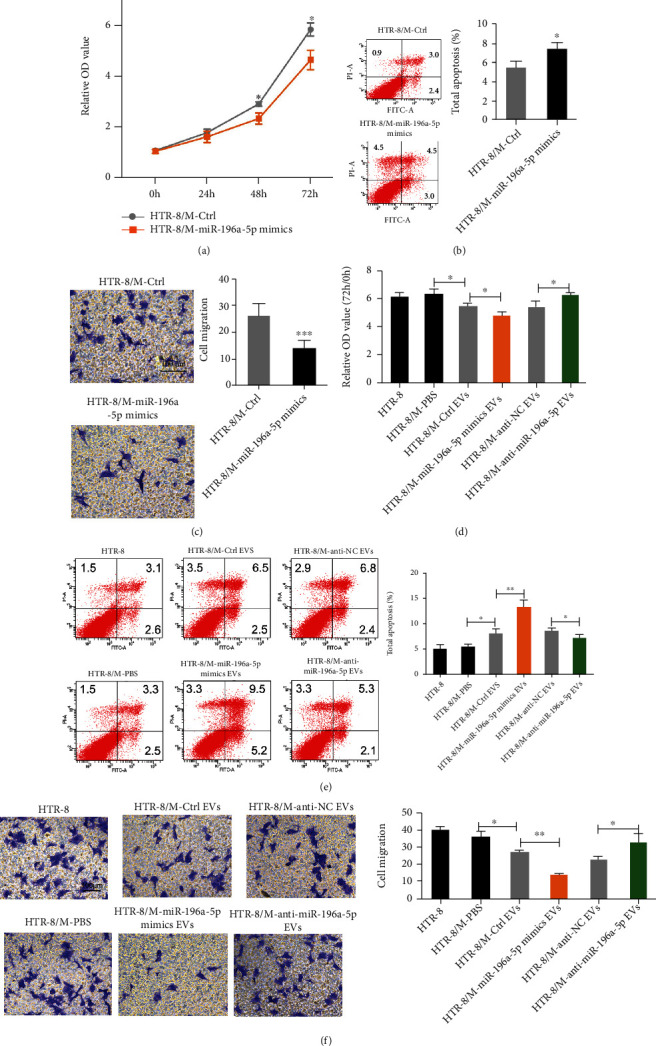
Trophoblast-derived EV miR-196a-5p induces M1 polarization of macrophages to suppress proliferation and invasion and promote apoptosis of HTR-8 cells. (a–c) PMA-pretreated THP-1 cells (M) were transfected with miR-196a-5p mimics and cocultured with HTR-8 cells. (a) CCK-8, (b) flow cytometry, and (c) Transwell assays were performed to analyze the proliferation, apoptosis, and migration of HTR-8 cells. (d–f) PMA-pretreated THP-1 cells were treated with EVs derived from miR-196a-5p overexpressing or miR-196a-5p knockdown HTR-8 cells and further cocultured with HTR-8 cells. (d) CCK-8, (e) flow cytometry, and (f) Transwell assays were performed to analyze the proliferation, apoptosis, and migration of HTR-8 cells (^∗^*p* < 0.05; ^∗∗^*p* < 0.01; ^∗∗∗^*p* < 0.001). Scale bar of Transwell assays were 100 *μ*m. EV: extracellular vesicle.

Numerous studies have shown an elevated M1 ratio affects trophoblast function in RM [26, 27]. Therefore, we established a coculture model to investigate the effects of M1 macrophages induced by trophoblast-derived EV miR-196a-5p on the proliferation, apoptosis, and migration of trophoblasts. As shown in Figures 6(a)–6(c), the proliferative and migratory abilities of HTR-8 cells were decreased, while the apoptotic potential was increased when cocultured with miR-196a-5p-overexpressing THP-1 cells (Figures 6(a)–6(c)). Additionally, PMA-pretreated THP-1 cells were incubated with PBS or EVs derived from miR-196a-5p overexpressing or knockdown HTR-8 cells or control cells and then cocultured with HTR-8 cells. We found that HTR-8 cells cocultured with PMA pretreated THP-1 cells (stimulated with EVs derived from control HTR-8 cells) exhibited reduced proliferation and migration and increased apoptosis (Figures 6(d)–6(f)). Similarly, the proliferative and migratory abilities of HTR-8 cells were decreased, whereas the apoptotic potential was increased when cocultured with PMA-pretreated THP-1 cells stimulated with miR-196a-5p-overexpressing HTR-8 cell-derived EVs (Figures 6(d)–6(f)). Inversely, the proliferative and migratory abilities of HTR-8 cells were increased, whereas the apoptotic potential was decreased when cocultured with PMA-pretreated THP-1 cells stimulated with miR-196a-5p knockdown HTR-8 cell-derived EVs (Figures 6(d)–6(f)). These results indicated that trophoblast-derived EV miR-196a-5p induces M1 polarization of macrophages, which in turn suppresses the proliferation and invasion and promotes apoptosis of HTR-8 cells.

### 3.7. M1 Macrophages Polarized by EV miR-196a-5p Regulate Cell Function of Trophoblasts via Secretion of TNF-*α*

**Figure 7:**
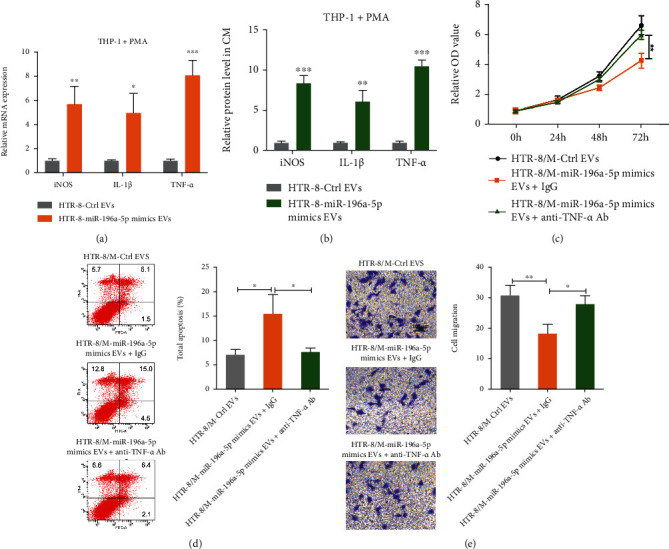
M1 macrophages polarized by EV miR-196a-5p regulate the function of trophoblasts via secretion of TNF-*α*. (a, b) PMA-pretreated THP-1 cells were treated with EVs derived from miR-196a-5p-overexpressing HTR-8 cells. (a) qPCR and (b) ELISA analysis of the mRNA and protein levels of iNOS, IL-1*β*, and TNF-*α*. (c–e) PMA-pretreated THP-1 cells were treated with EVs derived from miR-196a-5p-overexpressing HTR-8 cells and further cocultured with HTR-8 cells with a TNF-*α* antibody or IgG control antibody in the culture medium. (c) CCK-8, (d) flow cytometry, and (e) Transwell assays were performed to analyze proliferation, apoptosis, and migration of HTR-8 cells. Scale bar, 100 *μ*m (^∗^*p* < 0.05; ^∗∗^*p* < 0.01; ^∗∗∗^*p* < 0.001). EV: extracellular vesicle; PMA: phorbol 12-myristate 13-acetate.

A large number of studies have indicated that M1 macrophages affect the microenvironment of the maternal-fetal interface via secretion of cytokines [28, 29]. To investigate the mechanism of regulation of cell function of trophoblasts by M1 macrophages that had been polarized by EV miR-196a-5p, we performed qPCR and ELISA to analyze the levels of iNOS, IL-1*β*, and TNF-*α* in the cell lysates and CM of PMA-pretreated THP-1 cells. The results showed that both RNA and protein levels of TNF-*α*, iNOS, and IL-1*β* were increased after PMA-pretreated THP-1 cells were incubated with EVs derived from miR-196a-5p-overexpressing HTR-8 cells, and the increase in TNF-*α* levels was considerably greater than the increase in iNOS and IL-1*β* levels (Figures 7(a) and 7(b)). It has been reported that M1 macrophages inhibit trophoblast migration via TNF-*α* [29]. Therefore, we hypothesized that TNF-*α* mediated proliferation, migration, and apoptosis of HTR-8 cells via EV miR-196a-5p induced M1 macrophages. To verify this hypothesis, PMA-pretreated THP-1 cells were treated with EVs derived from miR-196a-5p-overexpressing HTR-8 cells and cocultured with HTR-8 cells with an anti-TNF-*α* antibody or IgG antibody added to the CM. CCK-8, flow cytometry, and Transwell assays demonstrated that the inhibition of proliferation and migration and promotion of apoptosis caused by EV miR-196-5p-induced M1 macrophages were partially attenuated by the anti-TNF-*α* antibody (Figures 7(c)–7(e)). Accordingly, we verified that M1 macrophage polarization by EV miR-196a-5p regulates cell function of trophoblasts via secretion of TNF-*α*.

## 4. Discussion

Physiological functioning of trophoblasts is essential for the implantation and development of maternal-fetal circulation. Dysregulation of the proliferative, migratory, and apoptotic capacities of trophoblasts in the maternal-fetal microenvironment plays a critical role in RM [30, 31]. Accumulating evidence has verified that miRNAs regulate trophoblast proliferation, migration, and apoptosis during pregnancy. It was reported that miR-196a-5p expression was decreased in the placental tissue of preeclampsia patients, and trophoblast migration could be restored under hypoxic conditions [32]. In this study, we showed that miR-196a-5p was upregulated in the CTBs of the villous tissues and plasma EVs of RM patients. Importantly, we demonstrated that EV miR-196a-5p affected trophoblast function by mediating the crosstalk between trophoblasts and macrophages. These findings may provide biomarker for predicting RM in the future.

Pregnancy maintenance depends on the immune tolerance and formation of a healthy placenta, both of which are involved in maternal-fetal communication between various types of cells, such as endothelial cells, trophoblasts, and immune cells [33, 34]. EVs can regulate the key signaling pathways between cell-to-cell interactions on maternal-fetal interface. For example, fetal cell-derived EVs promote inflammation in uterine and cervical cells, which resulted in labor and delivery [35]. Placenta-derived EVs play key roles in immune cell activation, differentiation, maturation, and endovascular homeostasis [36]. Here, we found that trophoblast-derived miR-196a-5p was packaged into EVs and transferred to macrophages with the assistance of the RNA-binding protein hnRNPA1, which specially binds to the UAGGUA sequence of miR-196a-5p. These results were consistent with another report that showed that the packaging of miR-196a-5p into EVs is mediated by hnRNPA1 in cancer-associated fibroblasts of head and neck cancer [22].

Macrophages are the second most abundant type of immune cells in the decidual microenvironment, and they functioned significant role of regulating the interactions on the maternal-fetal interface [19]. In general, decidual macrophages could be divided into classical activated (M1) and alternatively activated (M2) phenotypes, and appropriate regulation of the M1/M2 switch is essential for successful pregnancy [13]. Disturbances of the M1/M2 balance may cause pregnancy complications, such as RM, fetal growth restriction, and preeclampsia. It has been indicated that the polarization of macrophages is influenced by the local milieu of growth factors and cytokines [37]. Recently, studies revealed that EVs regulated M2 polarization of tumor-associated macrophages [20, 38]. However, it is unknown whether polarization of decidual macrophages could be induced by EVs. In our current study, we demonstrated that trophoblast-derived EV miR-196a-5p could induce M1 polarization of macrophages. This is consistent with other report that EV-derived miRNAs could be transferred into target cells and regulate these cells' biology [39]. Notably, this is the first study to show that trophoblasts could promote M1 polarization of macrophages via transfer of EV miRNA. Numerous studies have indicated that miRNAs suppress their target genes' expression via binding to their 3′-UTR [26, 40]. Here, we found that EV miR-196a-5p downregulated I*κ*B*α* expression and activated the NF-*κ*B pathway via binding to the 3′-UTR of I*κ*B*α*. Consequently, this study revealed a novel mechanism of M1 polarization of macrophages.

The ratio of M1 macrophages in the decidual tissue of unexplained RM patients was significantly increased compared with individuals who had a healthy pregnancy in the first trimester [14]. It has been reported that M1 macrophages play important roles in regulating trophoblast function via secretion of cytokines [28]. It has been reported that TNF inhibitors could reduce the immune rejection rate and improve the pregnancy outcomes in females suffering from RM [41]. In this study, we also found that EV miR-196a-5p-induced M1 macrophages could inhibit the proliferation and migration, while promoting the apoptosis of trophoblasts via TNF-*α*. This is consistent with the fact that M1 macrophages suppress trophoblast migration and invasion via TNF-*α* and IL-1 [29].

## 5. Conclusions

We discovered that miR-196a-5p expression was higher in CTBs and plasma EVs of RM patients. EV miR-196a-5p derived from trophoblasts could be transferred to macrophages, and this was mediated by hnRNPA1, and miR-196a-5p subsequently promoted M1 polarization of macrophages via downregulating I*κ*B*α* expression and activating NF-*κ*B signaling pathway. M1 macrophages induced by EV miR-196a-5p could suppress the proliferation and migration, while promote the apoptosis of trophoblasts via secretion of TNF-*α*. Thus, this study revealed a novel regulating mechanism underlying the pathogenesis of RM by identifying a novel means of crosstalk between trophoblasts and macrophages, which may contribute to the development of the preventative and therapeutic strategies for management of RM.

## Data Availability

The datasets during and/or analyzed during the current study are available from the corresponding authors on reasonable request.

## Supplementary Materials

Supplementary 1 Supplementary File 1: sequences of primers used for qPCR.

Supplementary 2 Supplementary File 2: details of the antibodies used in this study.

Supplementary 3 Supplementary File 3: the sequences of the primers used for plasmid construction and the sequences of the siRNAs used in this study.
