# Supplementary material for: miR-196a-5p-Rich Extracellular Vesicles from Trophoblasts Induce M1 Polarization of Macrophages in Recurrent Miscarriage
Source: J Immunol Res. 2022 May 23;2022:6811632. doi: 10.1155/2022/6811632 (PMC9153387; doi:10.1155/2022/6811632)
Supplement: Supplementary 1 — Supplementary File 1: sequences of primers used for qPCR. [file 6811632.f1.docx]

**Table S1. Sequences of primers used for qPCR.**

| miR-196a-5p | forward 5’- CCGACGTAGGTAGTTTCATGTT -3' |
| --- | --- |
|  | reverse 5’- GTGCAGGGTCCGAGGTATTC -3’ |
| CD68 | forward 5’- GGAAATGCCACGGTTCATCCA -3' |
|  | reverse 5’- TGGGGTTCAGTACAGAGATGC -3’ |
| CD80 | forward 5’- CCTCTCCATTGTGATCCTGG-3' |
|  | reverse 5’- GGCGTACACTTTCCCTTCTC-3’ |
| CD86 | forward 5’-GCACGTCTAAGCAAGGTCAC-3’ |
|  | reverse 5’-CATATGCCACACACCATCCG-3’ |
| iNOS | forward 5’-GTTCCAGATGAATACTGGCAGTC-3' |
|  | reverse 5’-GTTCCAGATGAATACTGGCAGTC-3’ |
| IL-1β | forward 5’-ATGATGGCTTATTACAGTGGCAA -3' |
|  | reverse 5’-GTACGGGATTGCCCCTCTG-3’ |
| TNF- *α* | forward 5’- GTGACAAGCCTGTAGCCCAT-3' |
|  | reverse 5’- CAGACTCGGCAAAGTCGAGA-3’ |
| CD163 | forward 5’- TTTGGACAAGCCGTGACTAGA -3' |
|  | reverse 5’- CATTCCCGGTGTTGACATTCC -3’ |
| CD206 | forward 5’- GGGTTGCTATCACTCTCTATGC -3' |
|  | reverse 5’- TTTCTTGTCTGTTGCCGTAGTT -3’ |
| Arginase-1 | forward 5’- GTGGAAACTTGCATGGACAAC -3' |
|  | reverse 5’- AATCCTGGCACATCGGGAATC -3’ |
| IL-10 | forward 5’- CCTCCGTCTGTGTGGTTTGAA -3' |
|  | reverse 5’- CACTGCGGTAAGGTCATAGGA -3’ |
| hnRNPA1 | forward 5’-TTTGGCGGTGGTAGTGGAAG-3' |
|  | reverse 5’-CTGGCTCTCCTCTCCTGCTA-3’ |
| IκBα | forward 5’- CATCCTGAAGGCTACCAACTAC -3' |
|  | reverse 5’- CATCAGCACCCAAGGACAC -3’ |
| U6 | forward 5’- GCTTCGGCAGCACATATACTAAAAT -3' |
|  | reverse 5’- CGCTTCACGAATTTGCGTGTCAT -3’ |
| GAPDH | forward 5’-TACTAGCGGTTTTACGGGCG-3' |
|  | reverse 5’-TCGAACAGGAGGAGCAGAGAGCGA-3’ |
