# Supplementary material for: miR-196a-5p-Rich Extracellular Vesicles from Trophoblasts Induce M1 Polarization of Macrophages in Recurrent Miscarriage
Source: J Immunol Res. 2022 May 23;2022:6811632. doi: 10.1155/2022/6811632 (PMC9153387; doi:10.1155/2022/6811632)
Supplement: Supplementary 2 — Supplementary File 2: details of the antibodies used in this study. [file 6811632.f2.docx]

**Table S2:** Details of the antibodies used in this study.

| **Antibody** | **WB** | **Flow Cyt** | **RIP** | **Specificity** | **Company** |
| --- | --- | --- | --- | --- | --- |
| Tsg101 (ab125011) | 1:1000 | - | - | Rabbit monoclonal | Abcam |
| CD9  (ab236630) | 1:1000 | - | - | Rabbit monoclonal | Abcam |
| β-actin  (ab8226) | 1:1000 | - | - | Mouse monoclonal | Abcam |
| CD86  (FAB141R100U) | - | 0.25μg/10^6^ cells | - | Mouse  monoclonal | R&D |
| Histone H3  (ab1791) | 1:5000 | - | - | Rabbit monoclonal | Abcam |
| hnRNPA1 (#8443) | 1:1000 | - | 1:100 | Mouse monoclonal | Cell Signaling Technology |
| Histone H3 (ab1791) | 1:1000 | - | - | Rabbit polyclonal | Abcam |
| Alix  (ab117600) | 1:500 | - | - | Mouse monoclonal | Abcam |
| p65  (#8242) | 1:1000 | - | - | Rabbit monoclonal | Cell Signaling Technology |
| IκBα  (ab32518) | 1:1000 | - | - | Rabbit monoclonal | Abcam |
