# Supplementary material for: miR-196a-5p-Rich Extracellular Vesicles from Trophoblasts Induce M1 Polarization of Macrophages in Recurrent Miscarriage
Source: J Immunol Res. 2022 May 23;2022:6811632. doi: 10.1155/2022/6811632 (PMC9153387; doi:10.1155/2022/6811632)
Supplement: Supplementary 3 — Supplementary File 3: the sequences of the primers used for plasmid construction and the sequences of the siRNAs used in this study. [file 6811632.f3.docx]

| **Name** | **sequence** |
| --- | --- |
| IκBα plasmid | Sense5’-CCGCTCGAGCGGCTCACCTTTGTGGGGTTTTT -3’ |
|  | Antisense5’CCGGAATTCCGGGTACACCATTTACAGGAGGG -3’ |
| siRNA-hnRNPA1-1 | Sense 5’-CAGCUGAGGAAGCUCUUCAdTdT-3’ |
|  | Antisense 5’-UGAAGAGCUUCCUCAGCUGdTdT-3’ |
| siRNA-hnRNPA1-2 | Sense 5’-GCCGAAGAAGCAUCGUUAAAGdTdT-3’ |
|  | Antisense 5’-UUAACGAUGCUUCUUCGGCGGdTdT-3’ |

**Table S3. The sequences of the primers used for plasmid construction, and the sequences of the siRNAs used in this study.**
