# Supplementary material for: miR-196a-5p-Rich Extracellular Vesicles from Trophoblasts Induce M1 Polarization of Macrophages in Recurrent Miscarriage
Source: J Immunol Res. 2022 May 23;2022:6811632. doi: 10.1155/2022/6811632 (PMC9153387; doi:10.1155/2022/6811632)
Supplement: Supplementary 4 — Supplementary File 4: supplementary figures and figure legends. [file 6811632.f4.pdf]

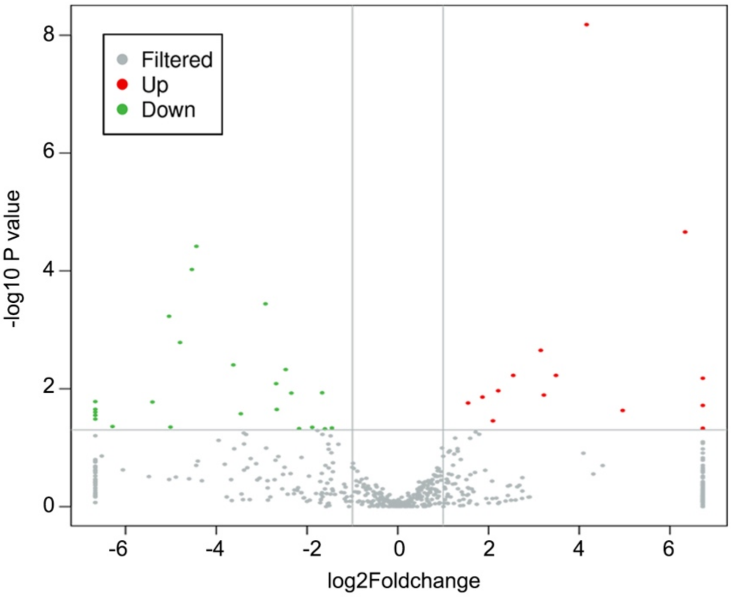

Figure S1 Differential expression analysis based on miRNA-seq of three RM and three healthy control cases. Volcano plot showing the differentially expressed miRNAs between the villous tissues of three RM and three healthy control cases. RM, recurrent miscarriage.

Score	Relative Score	RBP Name	Start	End	Matching sequence
7.7798874	75%	ZRANB2	2	7	AGGUAG
7.1355048	72%	HNRNPA1	1	6	UAGGUA
4.40359056	100%	ELAVL1	7	10	GUUU

(a)

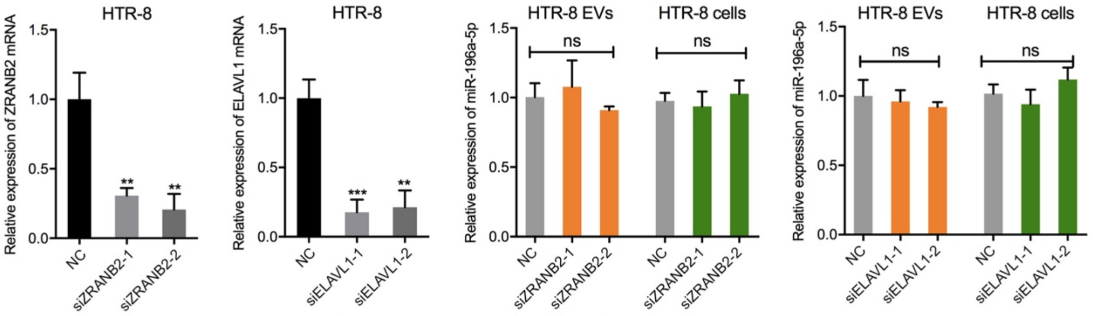

(b) (c) (d) (e)

Figure S2 Predicted RNA binding proteins associated with miR-196a-5p. (a) Predicted RNA-binding proteins according the database of RBP specificities (RBPDB, threshold 0.7). (b-c) The mRNA expression levels of ZRANB2 or ELAVL1 were measured using RT-qPCR in HTR-8 cells transfected with siZRANB2 or siELAVL1.

(d-e) qPCR analysis of miR-196a-5p expression levels in HTR-8 cell derived-EVs and the HTR-8 cells after transfection with siZRANB2 or siELAVL1. (**$p < 0.01$; ***$p < 0.001$; ns not significant).

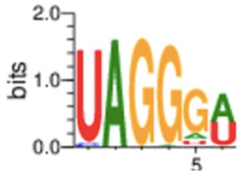

Figure S3 Predicted hnRNPA1 binding site on miR-196a-5p. The schematic diagram of hnRNPA1 binding site on miR-196a-5p.

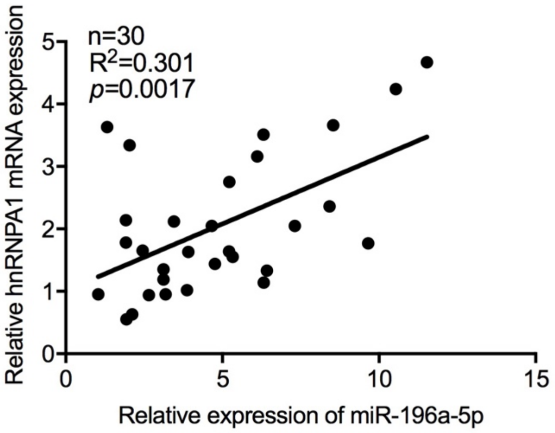

Figure S4 The expression of hnRNPA1 was positively associated with miR-196a-5p in 30 cases RM villous tissues. The correlation analysis between the expression of hnRNPA1 mRNA and miR-196a-5p in 30 cases RM villous tissues.

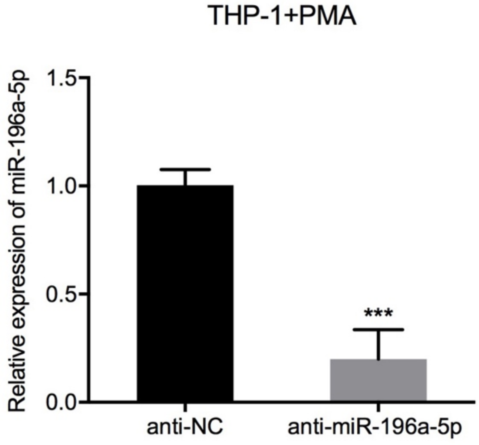

Figure S5 miR-196a-5p was downregulated in PMA-pretreated THP-1 cells after transfection with anti-miR-196a-5p. qPCR analysis of the expression of miR-196a-5p in PMA-pretreated THP-1 cells after transfection with anti-miR-196a-5p. PMA, phorbol 12-myristate 13-acetate.
